# Genome-wide expression analysis of novel heat-responsive microRNAs and their targets in contrasting wheat genotypes at reproductive stage under terminal heat stress

**DOI:** 10.3389/fpls.2024.1328114

**Published:** 2024-04-10

**Authors:** Monika Saroha, Aditi Arya, Gyanendra Singh, Pradeep Sharma

**Affiliations:** ^1^ Department of Biotechnology, ICAR Indian Institute of Wheat and Barley Research, Karnal, Haryana, India; ^2^ Department of Biotechnology, Deenbandhu Chhotu Ram University of Science and Technology, Murthal, Haryana, India

**Keywords:** *Triticum aestivum*, degradome, developing seed, flag leaf, heat stress, miRNA, wheat

## Abstract

**Introduction:**

Heat stress at terminal stage of wheat is critical and leads to huge yield losses worldwide. microRNAs (miRNAs) play significant regulatory roles in gene expression associated with abiotic and biotic stress at the post-transcriptional level.

**Methods:**

In the present study, we carried out a comparative analysis of miRNAs and their targets in flag leaves as well as developing seeds of heat tolerant (RAJ3765) and heat susceptible (HUW510) wheat genotypes under heat stress and normal conditions using small RNA and degradome sequencing.

**Results and discussion:**

A total of 84 conserved miRNAs belonging to 35 miRNA families and 93 novel miRNAs were identified in the 8 libraries. Tae-miR9672a-3p, tae-miR9774, tae-miR9669-5p, and tae-miR5048-5p showed the highest expression under heat stress. Tae-miR9775, tae-miR9662b-3p, tae-miR1120a, tae-miR5084, tae-miR1122a, tae-miR5085, tae-miR1118, tae-miR1130a, tae-miR9678-3p, tae-miR7757-5p, tae-miR9668-5p, tae-miR5050, tae-miR9652-5p, and tae-miR9679-5p were expressed only in the tolerant genotype, indicating their role in heat tolerance. Comparison between heat-treated and control groups revealed that 146 known and 57 novel miRNAs were differentially expressed in the various tissues. Eight degradome libraries sequence identified 457 targets of the differentially expressed miRNAs. Functional analysis of the targets indicated their involvement in photosynthesis, spliceosome, biosynthesis of nucleotide sugars and protein processing in the endoplasmic reticulum, arginine and proline metabolism and endocytosis.

**Conclusion:**

This study increases the number of identified and novel miRNAs along with their roles involved in heat stress response in contrasting genotypes at two developing stages of wheat.

## 1 Introduction

Wheat (*Triticum aestivum* L.) is a globally important crop that accounts for approximately 30% of worldwide cereal production. Being a thermo-sensitive crop, wheat requires a low temperature period for its physiological development, flowering and grain setting. An increase in 1°C temperature leads to a steep decline in wheat grain weight and nearly 6% decrease in wheat production (Joshi et al., 2007; Lobell et al., 2008; Chauhan et al., 2009; Asseng et al., 2015). Heat stress can affect various phenological stages of wheat by disruption of membranes and metabolic imbalance of protein structures caused by misfolding, unfolding and aggregation, thereby unbalancing the cell structure or metabolic and biochemical pathways (Sharma et al., 2010). These metabolic alterations adversely affect plant growth and various physiological and developmental processes like photosynthesis, respiration, reproduction, grain filling, and hence yield (Kaushal et al., 2016). However, there exists a wide diversity of wheat genotypes that have the innate potential to perceive heat stress and trigger the defense mechanism against it. This potential varies depending on the genotype. Also, the level of damage is significantly determined by the phenophase in which the plants are stressed (Porter and Gawith 1999). During flowering and grain filling, the crop is frequently exposed to brief periods of high warmth (33- 40°C), which often causes accelerated growth, flowering, and ripening (Rahman et al., 2009) and overall reduced yield.

Wheat plant photosynthetic activity is directly influenced by high temperature. Heat stress leads to the breakdown of the chlorophyll content in leaves during anthesis and grain filling, which in turn declines both leaf photosynthetic activity and final biomass (Liu et al., 2017).The flag leaf contributes to 58% of total photosynthesis (Hengyong and Junshi, 1995) resulting in the synthesis of 43% of the carbohydrates required for grain filling (Sharma et al., 2003), thereby playing an important role in total wheat production. Heat stress can lead to reduced photosynthesis in leaves because of the loss of photoreductive activity in chloroplasts (Hu et al., 2020). This in turn impacts the developing seeds too. These developing seeds undergo various morphological transformations and build-up of metabolites mainly starch and proteins till desiccation of aleurone and embryonic tissues is reached (Sabelli and Larkins, 2009). Starch contributes to about 75% of grain weight. Exposure to heat stress during the grain filling stage hinders the activity of proteins involved in the starch synthesis pathway, thereby reducing the amount of starch synthesis. This causes shrinkage in the grain due to ultrastructural changes in the aleurone layer and endosperm cells, hence reducing the grain weight and quality (Yamakawa and Hakata, 2010; Liao et al., 2014).

miRNAs represent a diverse class of 21-22 nucleotides long, non-coding small RNAs that not only regulate growth and development but also participate in various hormonal and metabolic pathways involved in environmental stress adaptation in plants. These miRNAs control a spectrum of biological development of leaves, roots, flowers etc. by either slicing or blocking the translation of their target mRNAs in a spatio-temporal way (Chen, 2010). Many studies have revealed the miRNome with the associated target genes during seed development stages in wheat (Li et al., 2015), maize (Kang et al., 2012), brassica (Wei et al., 2018), rice (Peng et al., 2011; Huang J. et al., 2016; Zhao et al., 2017), barley (Curaba et al., 2012). Due to polyploid complexity, repeat content and the big size of the wheat genome, many miRNAs and their corresponding targets are yet to be revealed (Budak and Akpinar, 2015).In wheat, miRNA studies have been done on diverse tissues at various developmental phases, resulting in the identification and estimation of the expression levels of many abiotic stress related conserved and novel miRNAs (Kantar et al., 2011; Tang et al., 2012; Li et al., 2013; Meng et al., 2013; Han et al., 2014; He et al., 2021; Zhou et al., 2019). Few studies have used a single wheat genotype to identify miRNAs differentially expressed in response to heat (Kumar et al., 2015; Li et al., 2015; Ragupathy et al., 2016; Ravichandran et al., 2019; Wang et al., 2019). However, very limited studies have been done work on contrasting genotypes at different development stages (Goswami et al., 2014; Liu et al., 2020). In this study, we identified heat stress related differentially expressed conserved as well as novel miRNAs and their target genes using small RNA sequencing. Eight sRNA libraries comprising of two contrasting wheat genotypes (heat-tolerant and heat-susceptible) in the two most vital organs in wheat grain-filling processes i.e. flag leaf and developing seeds at 10 days after anthesis at control and heat-stress conditions were sequenced. Using 8 libraries of degradome sequencing, we validated miRNA targets and their degradation sites. The results provide a comparative analysis of changes in the miRNA profiles and their targets in different wheat tissues and in genotypes with diverse genetic backgrounds and contrasting heat tolerances.

## 2 Materials and methods

### 2.1 Plant materials, stress treatments and collection of tissues

Seeds of two contrasting genotypes i.e., RAJ3765 (heat tolerant hereafter designated as HT) and HUW510 (heat sensitive hereafter designated as HS) were obtained from the Germplasm Section of ICAR-Indian Institute of Wheat and Barley Research, Karnal, India. Three seeds of each genotype were sown in 20 cm plastic pots under standard growth conditions. A total of 40 pots (20 for each genotype) were sown till the flag leaf stage was achieved. At the flag leaf stage, one set (5 pots) of the plants was kept as control (US) while the other set was treated with a heat treatment of 37°C (day)/27°C (night) for 24 hours (TCP). Flag leaves (FL) were harvested from all the pots of the particular set and pooled; flash-frozen and ground in liquid nitrogen for miRNA isolation. A similar treatment was given to another set of pots at the 10DAA stage (DA). For the developing grain stage, the main tiller spike of each wheat plant was sampled. Five spikelets from the middle of spikes from all the plants were harvested and immediately flash-frozen in liquid nitrogen and stored at -80°C till nucleic acid extraction. The spikelets were carefully taken out of the liquid nitrogen without thawing; developing seeds were separated and ground to a very fine powder using a sterile pestle and mortar in liquid nitrogen. The samples were referred to as PSTCPFL1 and PSTCPDA1 for heat-stressed flag leaf and developing seeds of RAJ3765 genotype; PSTCPFL2 and PSTCPDA2 for heat-stressed flag leaf and developing seeds of HUW510; PSUSFL1 and PSUSDA1 for control flag leaf and developing seeds of RAJ3765; PSUSFL2 and PSUSDA2 for control flag leaf and developing seeds of HUW510, respectively.

### 2.2 Small RNA library construction and sequencing

Total RNA of the samples was isolated in triplicates with TRIzol reagent (Invitrogen, USA) according to the manufacturer’s instructions. The purity and quantity of the isolated RNA samples were analyzed using Agilent Bioanalyzer (Agilent Technologies, U.S.A). RNA samples with integrity number ≥7.0 were further separated electrophoretically on 15% Tris-Borate-EDTA (TBE) urea denaturing polyacrylamide gel and the 18-30ntlong small RNA fraction was purified and further eluted. Equal amounts of RNA samples were isolated in triplicates from each sample and pooled together before proceeding to sequencing. 5’ and 3’ adaptors were sequentially ligated by T4 RNA ligase to the fractioned sRNA molecules and then reverse-transcribed to cDNA by RT-PCR. These cDNA samples were used as templates for double-stranded cDNA synthesis by PCR amplification using primers that annealed to adapters. The prepared libraries were quantified using a Qubit Fluorometer and validated for quality by running an aliquot on a Sensitivity Bio-analyser Chip (Agilent). The resulting libraries were sequenced using the Illumina Hiseq platform. The raw data have been submitted to NCBI and the accession number is PRJNA1012670.

### 2.3 Identification of conserved and novel miRNAs and their targets

The raw reads obtained from the libraries were processed by removing 3’ TruSeqadaptors, a minimum of 8 characters were checked for. The adapter trimmed sequences were filtered based on low-quality reads and length (min length 16 and max. length 40) by using FASTQ/A Clipper of FASTX-Toolkit, version-0.0.13(http://hannonlab.cshl.edu/fastx_toolkit/index.html).The filtered reads were BLASTn against miRBase 22 (http://www.miRBase.org, accessed on June 2022). An E-value cut-off of 0.001 was used. The miRNAs that aligned to previously reported miRNAs were designated as known miRNAs. The sequences that did not align to miRBase and the reads filtered due to length and 3’ adapter prior to aligning to miRBase were referred to as un-annotated sequences. These were used for novel miRNA prediction. These un-annoated sequences were given as input along with mapping (BED) file containing the annotation tracks and wheat genome assembly, RefSeq v2.0 (https://wheat-urgi.versailles.inra.fr/Seq-Repository/Assemblies) to Mireap v0.22b (http://sourceforge.net/projects/mireap). The Mireap program was run with following parameters: Minimal miRNA sequence length (18); Maximal miRNA sequence length (26); Minimal miRNA reference sequence length (20); Maximal miRNA reference sequence length (24); Maximal free energy allowed for a miRNA precursor (−20 kcal/mol); Minimal base pairs of miRNA and miRNA* (14); Maximal bulge of miRNA and miRNA* (3); Maximal asymmetry of miRNA/miRNA* duplex (2); Flank sequence length of miRNA precursor (10). Mireap identified novel miRNAs based on alignment, secondary structure, free energy and location on the precursor arm. The secondary structures of novel miRNAs were predicted by RNAfold (http://rna.tbi.univie.ac.at/cgi-bin/RNAWebSuite/RNAfold.cgi, accessed on August, 2022) using default parameters. The selection criterion for novel miRNAs was based on the stringent guidelines as described by Meyers et al. (2008). The major parameters were: (1) the precursor sequence was able to fold into a hairpin structure; (2) a mature miRNA sequence was located in one arm of the hairpin structure; (3) miRNAs contained less than six mismatches with the opposite miRNA* sequence on the other arm; (4) no loops or breaks in the miRNA* sequences; (5) the predicted secondary structures had greater minimal free energy indices (MFEIs) and negative minimum fold energies (MFEs); (6) the predicted mature miRNA sequence had no more than four mismatches as compared with its corresponding mature miRNA homologue.

### 2.4 Differential expression analysis of miRNAs

The expression patterns of miRNAs in different tissues were analyzed using DESeq package (v 1.12.1) (Anders and Huber, 2010). The number of reads mapping to each miRNA was normalized and the expression levels represented as RPM (Reads per Million) values of the total sRNA reads in the library. A t-test was applied to evaluate statistical significance. If the RPM ratios between the different pairwise comparisons were greater than 2 (fold change ≥ 2) and if the P-value was ≤0.05, then the miRNAs were defined as differentially expressed miRNAs.

### 2.5 Degradome library construction

Eight plant samples (namely PSTCPFL3 and PSTCPDA3 for heat-stressed flag leaf and developing seeds of RAJ3765; PSTCPFL4 and PSTCPDA4 for heat-stressed flag leaf and developing seeds of HUW510; PSUSFL3 and PSUSDA3 for control flag leaf and developing seeds of RAJ3765; PSUSFL4 and PSUSDA4 for control flag leaf and developing seeds of HUW510) were used to isolate total RNA. Briefly, 75-μg of Total RNA was taken for poly (A) enrichment (Dynabeads^®^ Oligo (dT)25-61002 protocol). 5’-end DNA adapter ligation was done overnight at 16°C. Unligated 5’-end adapters were removed by binding poly (A) enriched RNA on Dynabeads (dT)25. The Oligo(dT) enriched 5’-end adapters ligated mRNA was used as input for first strand synthesis, followed by 15 cycles of PCR to enrich adapter-ligated fragments (Denaturation at 95°C for 3 min, cycling (95°C for 20sec, 55°C for 30sec, 72°C for 60sec) and 72°C for 5mins). The PCR product was cleaned up using Hiprep beads (Magbio). The cleaned-up product was used for 12 cycles of Index PCR to add Nextera XT Index adapters (Denaturation at 95°C for 3 min, cycling (95°C for 20sec, 55°C for 30sec, 72°C for 60sec) and 72°C for 5mins). The final PCR product (sequencing library) was purified with HighPrep beads, followed by library quality control. The Illumina-compatible sequencing library was quantified by Qubit fluorometer (Thermo Fisher Scientific, MA, USA) and its fragment size distribution was analyzed on Agilent TapeStation. Finally, the sequencing library was accurately quantified by quantitative PCR using the Kapa Library Quantification Kit (Kapa Biosystems, Wilmington, MA, USA) and sequenced on an Illumina sequencer. The raw data have been submitted to NCBI and the accession number is PRJNA1013021.The raw reads (single-end; 50 bp) were processed using FASTQ/A Clipper of FASTX-Toolkit,version-0.0.13 (http://hannonlab.cshl.edu/fastxtoolkit/index.html) to remove the low-quality reads and adapters. The filtered reads were aligned to the Rfam database (version 14.8, (http://rfam.sanger.ac.uk, accessed in August, 2022) by using Bowtie2 version (2.3.4.3) to remove the noncoding RNAs, including rRNAs, tRNAs and snRNAs. The reads matching to rRNAs, tRNAs, snoRNAs and repeats were eliminated. The clean reads were mapped on wheat transcriptome (IWGSC v2.0) with a maximum of one mismatch using the Cleave-Land v4.4 pipeline (Addo-Quaye et al., 2008) to predict miRNA cleavage sites. Alignments with no mismatches at the cleave sites at the 10th position relative to the aligned miRNA and with a P-value ≤ 0.05 were considered as potential targets. Based on the relative degradome read abundance, the identified sites were divided into five categories (0-4). Categories 0-3 had more than one read and Category 4 had only one read mapped at the cleavage site. These categories showed the degree of prediction confidence; with Category 0 being the highest level of confidence and Category 4 represents the lowest level. T-plots were made according to the distribution of signatures (and abundances) along these transcripts so as to easily analyze the miRNA targets and RNA degradation patterns. The GO enrichment (Ashburner et al., 2000) and KEGG pathway analysis (Kanehisa et al., 2004) were performed on target genes of differentially expressed miRNAs to determine the function of the miRNA targets and their association with biological pathways using Blast2GO version 2.8 (https://www.blast2go.com/). Both the GO and the pathway enrichment analyses were performed at P-value with a cut-off of 0.05. To further investigate the association of heat-responsive miRNAs with their targets, we used the Cytoscape v3.9.1 platform (Shannon et al., 2003) to construct the miRNA-mediated regulatory networks.

### 2.6 Quantification by real-time PCR

miRNAs from the 8 samples were isolated using mirVana™ miRNA Isolation Kit (Ambion, USA) and reverse transcribed into cDNAs as per manufacturer’s instructions. Real-time expression levels of these miRNAs were done on BioRad CFX 96 (Biorad, UK) using miRNA-specific primers (**Supplementary Table 8**). For each reaction, 5 μl of 1:20 diluted template cDNA was mixed with 10 μl SYBR green PCR master mix 0.5 μl each of the forward and reverse primers and 4 μl of diethylpyrocarbonate (DEPC)-treated ddH_2_O were added to a final volume of 20 μl. The amplification program was as follows: initial denaturation at 95°C for 5 min, followed by denaturation for 15 s at 95°C and annealing for 15 s at 55°C for 40 cycles followed by melting at a temperature between 65°-95°C with an increment of 0.5°C for 10 s. Universal qPCR Primer was used as the reverse primer in the qPCR reactions. The fold change was calculated using a reference wheat U6 primer. All reactions of each sample were performed with three biological replicates. The data from different PCR runs on different cDNA samples were normalized to the mean Ct values of the internal standard gene. The comparative Ct (2^-ΔΔCt^) method was used for calculating the changes in miRNA expression level as a relative fold difference between an experimental and control sample (Pfaffl, 2001).

## 3 Results

### 3.1 Analysis of sRNA library datasets and sRNA profiles

**Figure 1:**
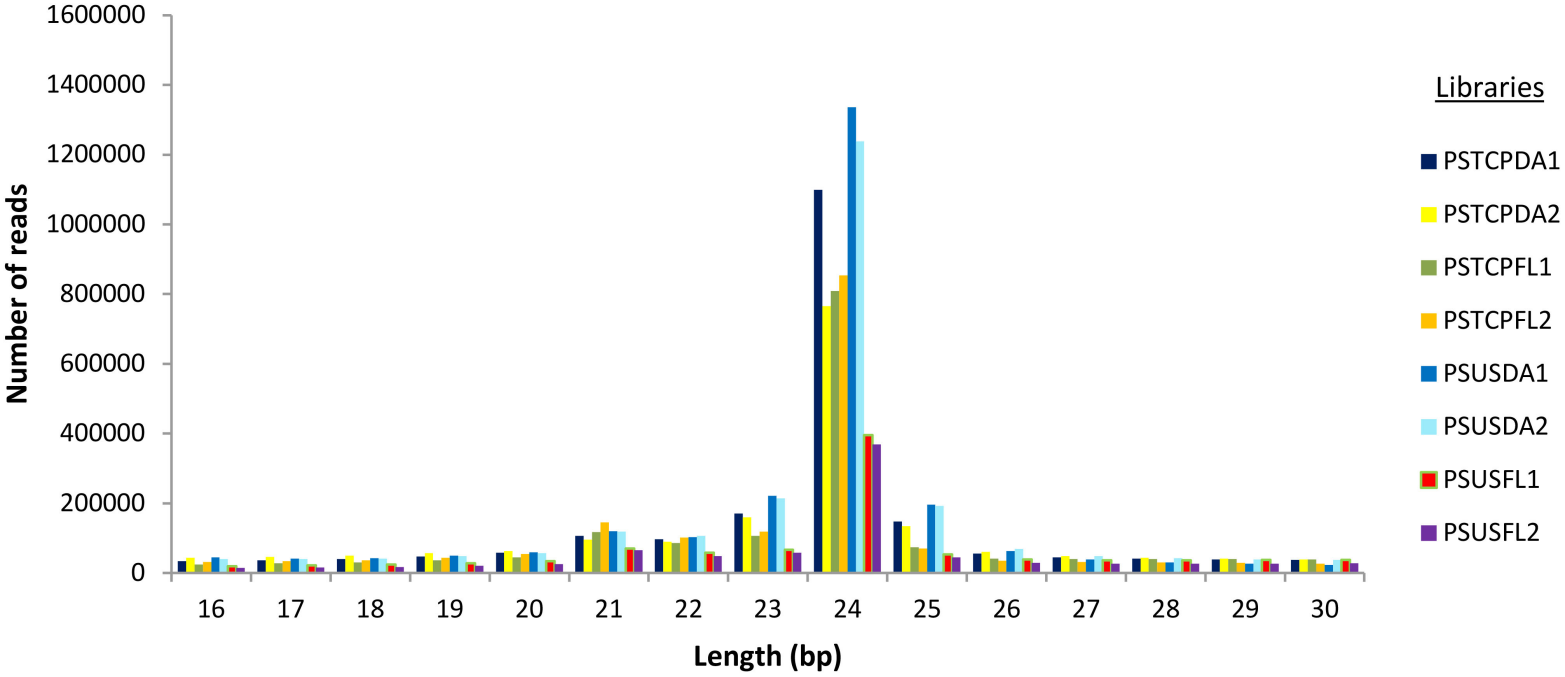
sRNA length distribution in the 8 libraries.

For the flag leaf and developing seed stages of the contrasting wheat genotypes under heat stress and non-stress conditions, eight sRNA libraries were constructed and sequenced via the Illumina HiSeq platform. Post-sequencing, the low-quality reads and adapter sequences were removed. We obtained a total of 21729502 reads (2347361 unique) for PSTCPDA1, 14114958 reads (unique 2536538) for PSUSDA1, 18148300 reads (2034161 unique) for PSTCPDA2,18880691 reads (2614016 unique) for PSUSDA2,17815732 reads (1858244 unique reads) for PSTCPFL1, 20675796 reads (1337389 unique) for PSUSFL1, 16604247 reads (1830757 unique) for PSTCPFL2 and 15100582 reads (1113537 unique) for PSUSFL2.The read statistics and percentage of various sRNAs (rRNA, snoRNA, snRNA, tRNA, miRNA) obtained are listed in **Table 1**. The proportion of sRNA length distribution in all 8 libraries varied from 16 to 26 nucleotides as shown in **Figure 1**, among which the 24 nt sRNA was the most abundant. The read statistics of the input files and sRNAs length distribution are provided in **Supplementary Table S1**.

**Table 1:** Summary of 8 small RNA libraries prepared from control and stressed flag leaf and developing seed of wheat genotypes RAJ3765 and HUW510.

Genotype, stage, condition	Library name	Total Reads	Trimmed Unique Reads	Reads aligned to genome	% Reads aligned to genome	rRNA	snoRNA	snRNA	tRNA	Clustered Reads	Reads aligned to mirBase	Known miRNA Uniq	Reads used for Novel miRNA	Reads utilized for Novel miRNA	Novel miRNA predicted
RAJ3765 10DAA stress	PSTCPDA1	21729502	2347361	2211250	94.2	248558	15447	5678	22	1418614	6539	104	1412075	1399975	170
HUW510 10DAA stress	PSTCPDA2	18148300	2034161	1900038	93.41	213089	19210	7557	67	1083136	3168	97	1079968	1068179	164
RAJ3765 FL stress	PSTCPFL1	17815732	1858244	1756395	94.52	278560	8137	3643	34	1080266	7700	102	1072566	1062425	215
HUW510 FL stress	PSTCPFL2	16604247	1830757	1739928	95.04	200800	11900	4227	37	1150245	9110	100	1141135	1129851	365
RAJ3765 10DAA Control	PSUSDA1	14114958	2536538	2415807	95.24	178700	9971	2552	52	1616834	6782	104	1610052	1595354	221
HUW510 10DAA Control	PSUSDA2	18880691	2614016	2457841	94.03	241611	12493	3197	42	1574858	7245	105	1567613	1551757	196
RAJ3765 FL Control	PSUSFL1	20675796	1337389	1263334	94.46	213984	8075	2494	15	654595	4705	98	649890	644115	172
HUW510 FL Control	PSUSFL2	15100582	1113537	1036175	93.05	179769	8148	2008	16	593561	4862	98	588699	582832	172

### 3.2 Identification of conserved miRNAs and potentially novel miRNAs

**Figure 2:**
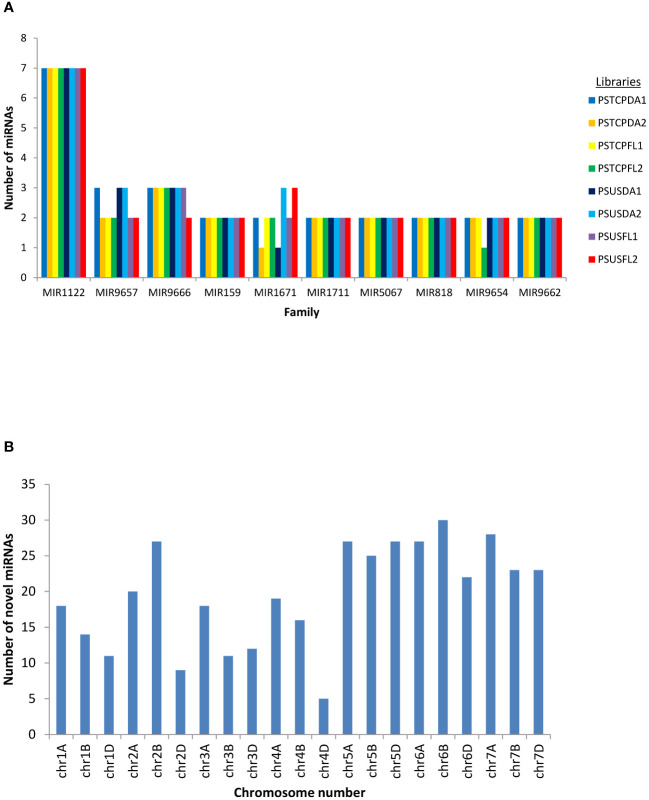
**(A)** miRNA family distribution of the known miRNAs among all the libraries **(B)** Chromosome-wise distribution of the novel miRNAs identified in all the libraries.

Conserved and novel miRNAs were identified by aligning the small RNA sequences from each library to the miRNAs available in miRBase. Finally, 424 known miRNAs belonging to 35 miRNA families were identified in the 8 libraries (**Supplementary Table S2**). The number of miRNAs varied among families. The most abundant family was represented by MIR1122 followed by MIR9657, MIR9666 and MIR159 (**Figure 2A**). MIR2275 was exclusively detected only in the seed libraries of both the genotypes, while the MIR1119 family was observed only in the seed libraries of HS genotype. 104 known miRNAs were identified in the stressed and non-stressed developing seeds of heat heat-tolerant genotype whereas 97 and 105 known miRNAs were detected in the developing seeds of the heat-susceptible genotype at stress and non-stress conditions (**Table 1**).

**Figure 3:**
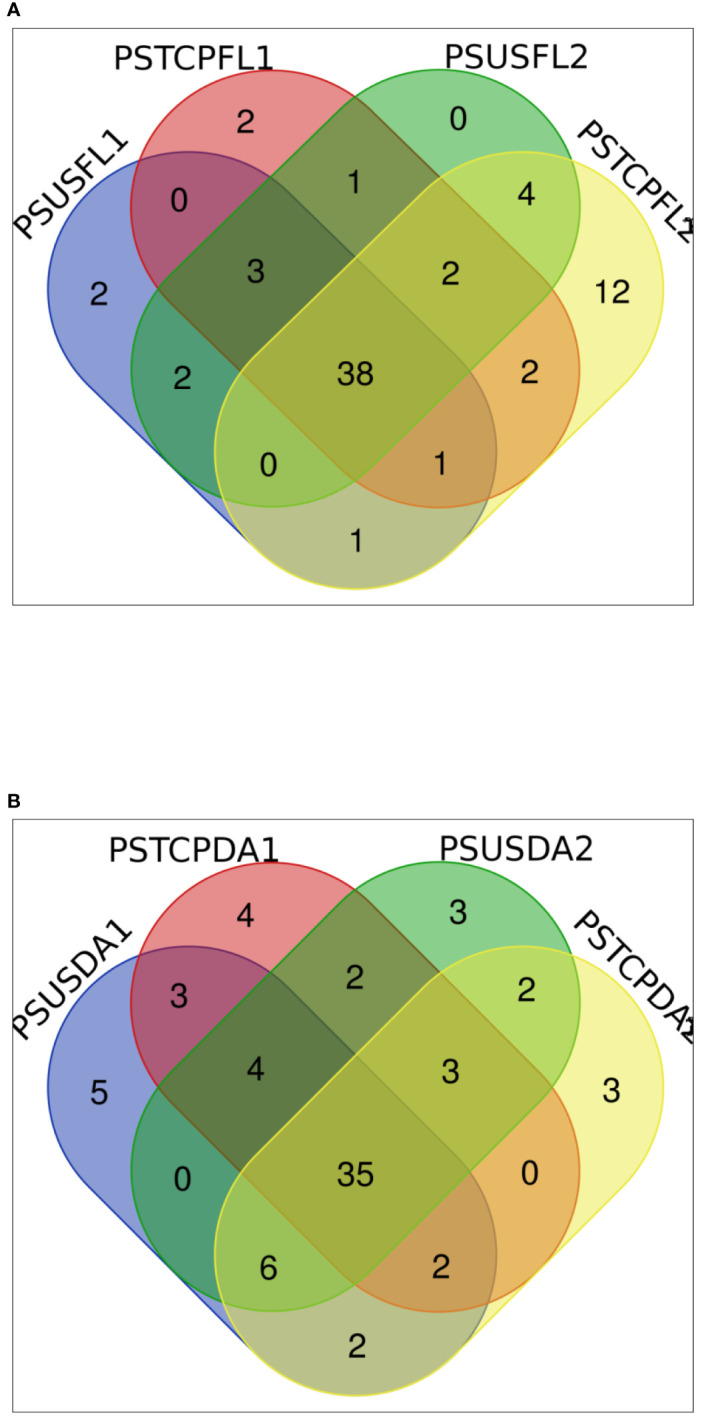
Distribution of the expressed known miRNAs in **(A)** flag leaves **(B)** developing seeds.

The distribution of miRNAs across libraries indicated that 38 known miRNAs were common to flag leaves of both sensitive and tolerant genotypes in the control and heat-treated samples (**Figure 3A**). Comparison of the genotypes separately indicated that 42 miRNAs were common between control and heat-stressed flag leaf samples of HT genotype, out of which 2 miRNAs such as tae-miR396-5p, tae-miR9670-3p were specific to flag leaf of the HT at control samples, whereas 7 miRNAs such as tae-miR9775, tae-miR9662b-3p, tae-miR1120a,tae-miR5084, tae-miR1122a, tae-miR7757-5p, and tae-miR5085 were expressed only in heat-treated flag leaf of the HT genotype (**Table 2**). In the HS genotype, 44 miRNAs were commonly expressed between control and heat treatments in the flag leaves. Only 6 miRNAs such as tae-miR1122a, tae-miR9772, tae-miR1121, tae-miR9679-5p, tae-miR1136, and tae-miR1120b-3p were expressed only in control conditions, while 16 miRNAs such as tae-miR9664-3p, tae-miR9657b-3p was expressed specifically after heat exposure (**Table 2**).

**Table 2:** MiRNAs specifically expressed in control and stress tissues of RAJ3765 and HUW510.

Control Conditions	Stress Conditions
**Expressed only in control RAJ3765 FL (PSUSFL1)** tae-miR396-5p, tae-miR9670-3p **Expressed only in control RAJ3765 developing seed (PSUSDA1)** tae-miR398, tae-miR1136, tae-miR9672b, tae-miR9670-3p, tae-miR9654a-3p, tae-miR9664-3p, tae-miR167c-5p, tae-miR408, tae-miR164, tae-miR399, tae-miR531, tae-miR9657b-3p, tae-miR5384-3p **Expressed only in control HUW510 FL (PSUSFL2)** tae-miR1122a, tae-miR9772, tae-miR1121, tae-miR9679-5p,tae-miR1136, tae-miR1120b-3p **Expressed only in control HUW510 developing seed (PSUSDA2)** tae-miR9657a-3p, tae-miR396-5p, tae-miR1125, tae-miR7757-5p, tae-miR9668-5p, tae-miR9674b-5p, tae-miR9666b-5p, tae-miR9774, tae-miR1127a	**Expressed only in stressed RAJ3765 FL (PSTCPFL1)** tae-miR9775, tae-miR9662b-3p, tae-miR1120a, tae-miR5084, tae-miR1122a, tae-miR7757-5p, tae-miR5085 **Expressed only in stressed RAJ3765 developing seed (PSTCPDA1)** tae-miR1118, tae-miR1130a, tae-miR9678-3p, tae-miR9775, tae-miR7757-5p,tae-miR9668-5p, tae-miR5050, tae-miR9652-5p, tae-miR9679-5p **Expressed only in stressed HUW510 FL (PSTCPFL2)** tae-miR9664-3p, tae-miR9664-3p, tae-miR408, tae-miR530, tae-miR1135, tae-miR9666a-3p, tae-miR9779, tae-miR9676-5p, tae-miR9660-5p, tae-miR6201, tae-miR9673-5p, tae-miR6197-5p **Expressed only in stressed HUW510 developing seed (PSTCPDA2)** tae-miR2275-3p, tae-miR5084, tae-miR9779, tae-miR1135, tae-miR1847-5p, tae-miR160, tae-miR9666b-3p

In the developing seeds samples, 35 miRNAs were common among the four samples. Furthermore, control and heat libraries of the HT genotype shared 44 miRNAs, 13 miRNAs were expressed specifically in control conditions, while 9 miRNAs specific to heat-treated developing seeds in HT genotype (**Table 2**).

The control and heat treated libraries of the HS genotype developing seeds had 46 common miRNAs (**Table 2**, **Figure 3B**). Nine miRNAs showed specific expression in the control sample, whereas seven miRNAs expressed specifically in response to heat stress in the HS developing seeds (**Table 2**).

Potential novel miRNAs were identified using Mireap v0.2 based on the common criteria (as mentioned in methods). A maximum of 365 total novel miRNAs were identified in the PSTCPFL2 library while PSTCPDA2 represented only 164 total novel miRNAs. Of these 175 only 73 were detected with read count >=10. Chromosomal distribution indicated that the novel miRNAs were located on all wheat chromosomes with the maximum number on chromosomes 6B and 7A and a minimum on 4D (**Figure 2B**). Novel miRNAs are tentatively represented with the prefix ‘ps# number’. Detailed information on all the novel miRNAs in all 8 libraries, including the precursor sequences, precursor structures, chromosomal loci, read counts, + or – strand, and MFE values are provided in **Supplementary Table S3**. In addition, the secondary structures of a few novel miRNA precursors were predicted using RNAfold (http://rna.tbi.univie.ac.at/RNAfold) provided in **Supplementary Figure S1**.

### 3.3 Expression analysis of miRNAs

**Figure 4:**
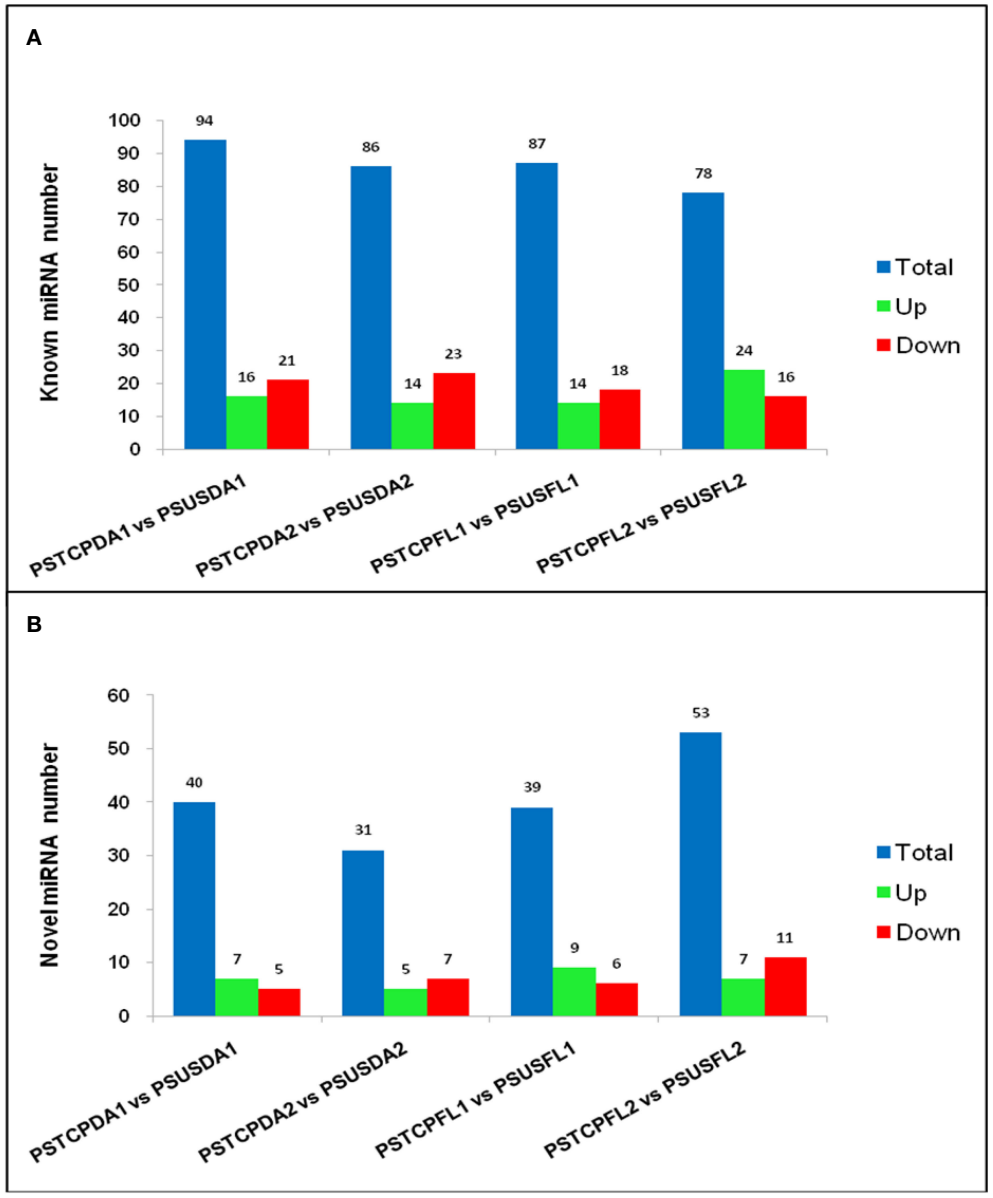
Summary of differentially expressed microRNAs. Regulation and number of known **(A)** and novel DEMs **(B)** in the different libraries.

**Figure 5:**
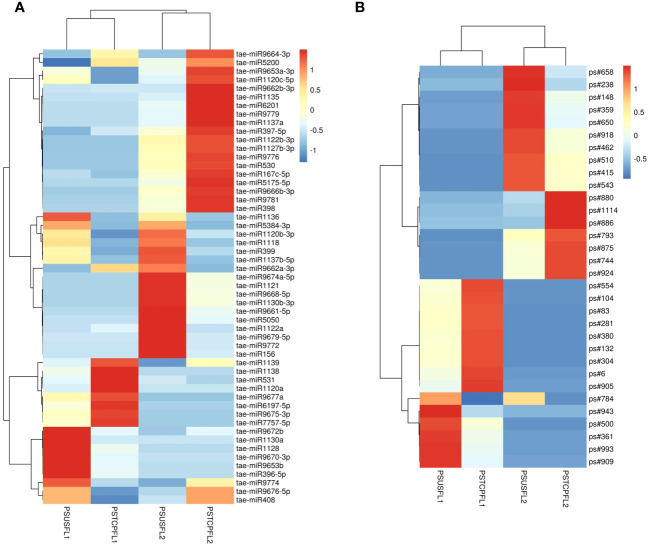
Heat map of the differentially expressed known **(A)** and novel **(B)** miRNAs in response to heat stress in flag leaves of the two genotypes. The dendrogram represents the hierarchical clustering of the control and heat treatments in these libraries.

**Figure 6:**
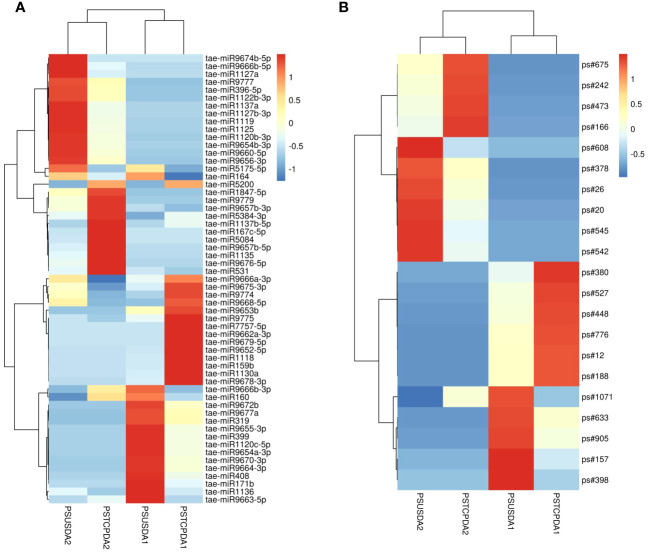
Heat map showing differentially expression of known miRNAs **(A)** and novel miRNAs **(B)** in response to heat stress in the developing seeds of the two wheat genotypes. The values shown on bars at the right side indicate fold change regulation in heat treated samples in comparison with control. The dendrogram represents the hierarchical clustering of the control and heat treatments in these libraries.

Comparisons between the normalized data of the heat-stressed and control flag leaf of the HT genotype RAJ3765 (PSTCPFL1 vs PSUSFL1)revealed that a total of 87known miRNAs were commonly expressed in both the samples, out of which 14 were up-regulated and 18 were down-regulated (**Supplementary Table S4**). Only 15 novel miRNAs showed differential expression in these libraries. The flag leaf of the HS genotype HUW510 (PSTCPFL2 vs PSUSFL2) showed a total of 78 miRNAs which were common in both control and heat-stressed samples. 24 known and 7 novel miRNAs were upregulated while 16 known and 11 novel miRNAs were downregulated in this library. A total of 94conserved and 40 novel miRNAs were commonly expressed in the developing seeds of control and heat-stressed RAJ3765 (PSTCPDA1 vs PSUSDA1), of which 16 conserved and 7 novel miRNAs were upregulated whereas 21known and 7 novel miRNAs were downregulated. HUW510 developing seeds (PSTCPDA2 vs PSUSDA2) showed 14 up and 23 down-regulated known miRNAs while 12 novel miRNAs were differentially expressed (**Figures 4A, B**). Hierarchical clustering as well as the heat maps of the differentially expressed known and novel miRNAs in response to heat in the flag leaf and developing seeds of both the genotypes is shown in **Figures 5**, **6**.

Some of the miRNAs showed contrasting expression in the two genotypes in response to heat stress e.g.in the developing seeds, tae-miR167c-5p, tae-miR9663-5p, tae-miR5084, tae-miR160, tae-miR1847-5p, tae-miR9666b-3p, tae-miR531 showed down-regulation in the HT and upregulation in the HS genotype respectively whereas tae-miR9775, tae-miR7757-5p, tae-miR9675-3p, tae-miR9774, tae-miR9666a-3p, tae-miR9668-5p were upregulated in the HT and downregulated in the HS genotype. Similarly, tae-miR1120c-5p, tae-miR9653a-3p, tae-miR9676-5p, tae-miR167c-5p, tae-miR9774, tae-miR9672b, tae-miR408 showed down regulation in the flag leaves of the HT and upregulation in the HS genotype.

### 3.4 Target gene functions of differentially expressed miRNAs

To ascertain the regulatory roles of the miRNAs, degradome sequencing of the 8 libraries was done to identify the target genes which resulted in more than 30M raw reads and above 11M reads in each library after filtering (**Supplementary Table S6**). The degradome libraries were designated as PSTCPFL3 and PSTCPDA3 for heat-stressed flag leaf and developing seeds of RAJ3765; PSTCPFL4 and PSTCPDA4 for heat-stressed flag leaf and developing seeds of HUW510; PSUSFL3 and PSUSDA4 for control flag leaf and developing seeds of RAJ3765; PSUSFL4 and PSUSDA4 for control flag leaf and developing seeds of HUW510 respectively.

**Figure 7:**
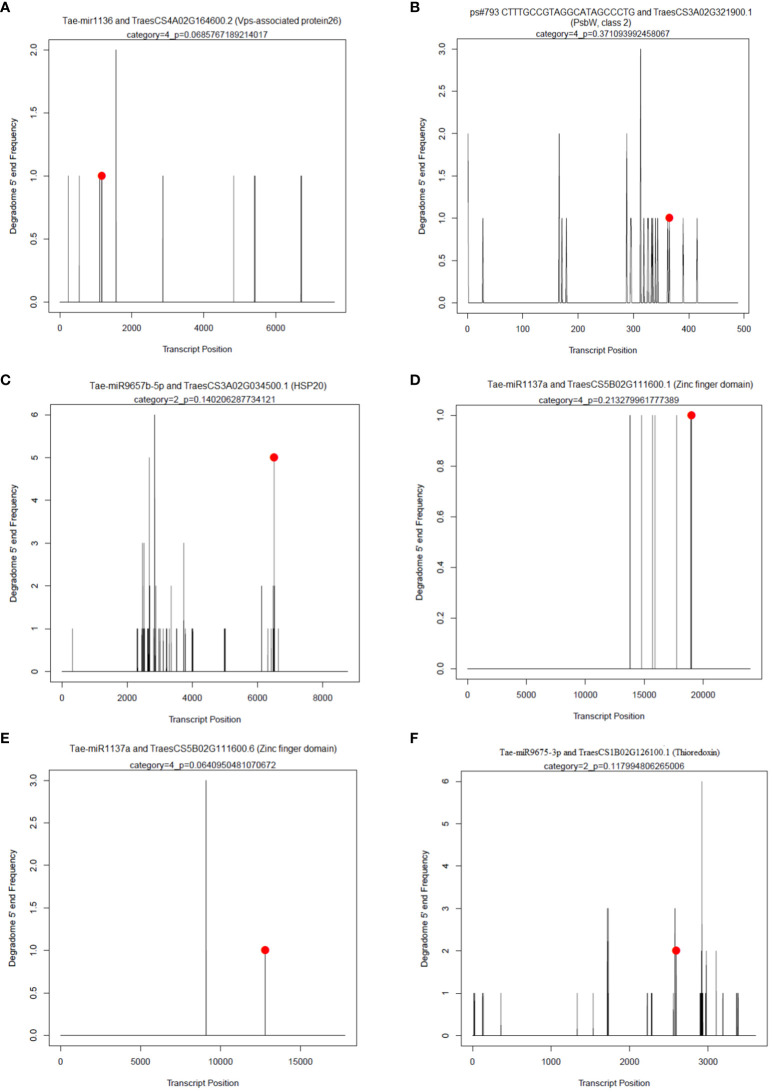
Target plots (T-plots) of miRNAs targets confirmed by degradome sequencing. In T-plots, the red dots indicate the miRNA-directed cleaved transcript. The X axis indicates the nucleotide position in target cDNA. The Y axis indicates the number of reads of cleaved transcripts detected in the degradome cDNA library. **(A)** Tae-mir1136 and TraesCS4A02G164600.2 (Vps-associated protein26) **(B)**; ps#793 CTTTGCCGTAGGCATAGCCCTG and TraesCS3A02G321900.1 (PsbW, class 2); **(C)** Tae-miR9657b-5p and TraesCS3A02G034500.1 (HSP20); **(D)** Tae-miR1137a and TraesCS5B02G111600.1 (Zinc finger domain); **(E)** Tae-miR1137a and TraesCS5B02G111600.6 (Zinc finger domain); **(F)** Tae-miR9675-3p and TraesCS1B02G126100.1 (Thioredoxin).

In each sample, an average of 43 non-redundant targets for known miRNAs and 91 targets for novel miRNAs with a P-value of ≤ 0.05 and a category of ≤ 4 were found. The maximum targets for known miRNAs were identified for PSUSDA4 (64) and the min for PSUSDA3 (36). Similarly, for novel miRNAs, PSTCPL4 (155) and PSUSFL4 (41) had maxi and min targets. The maximum cleavage sites belonged to Category 4 and the minimum to Category 1. Using degradome sequencing, 296 targets were identified for a total of 61 known miRNAs. The maximum targets were obtained for members of miR1137a family (51), followed by miR1127b-3p (32) and miR1136 (17). A few examples of the t-PLOTS are given in **Figure 7**.

**Figure 8:**
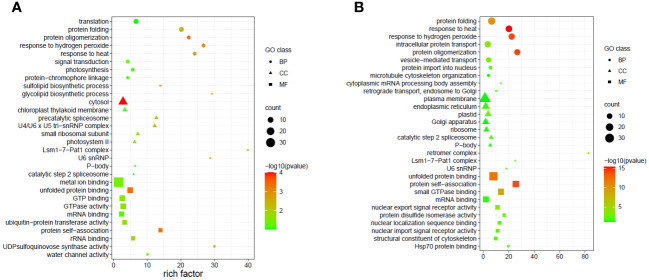
Top 30 enriched GO terms of the targets of the differentially expressed miRNAs in different tissues **(A)** Flag leaves and **(B)** Developing seeds of two wheat genotypes. The x-axis indicates the rich factor of such GO terms in each sample. The color of the point indicates the -log10 (p-value) of the GO terms, the size of the point indicates the number of genes enriched in such GO term, and the shape of the point indicates the domains of the GO terms.

**Figure 9:**
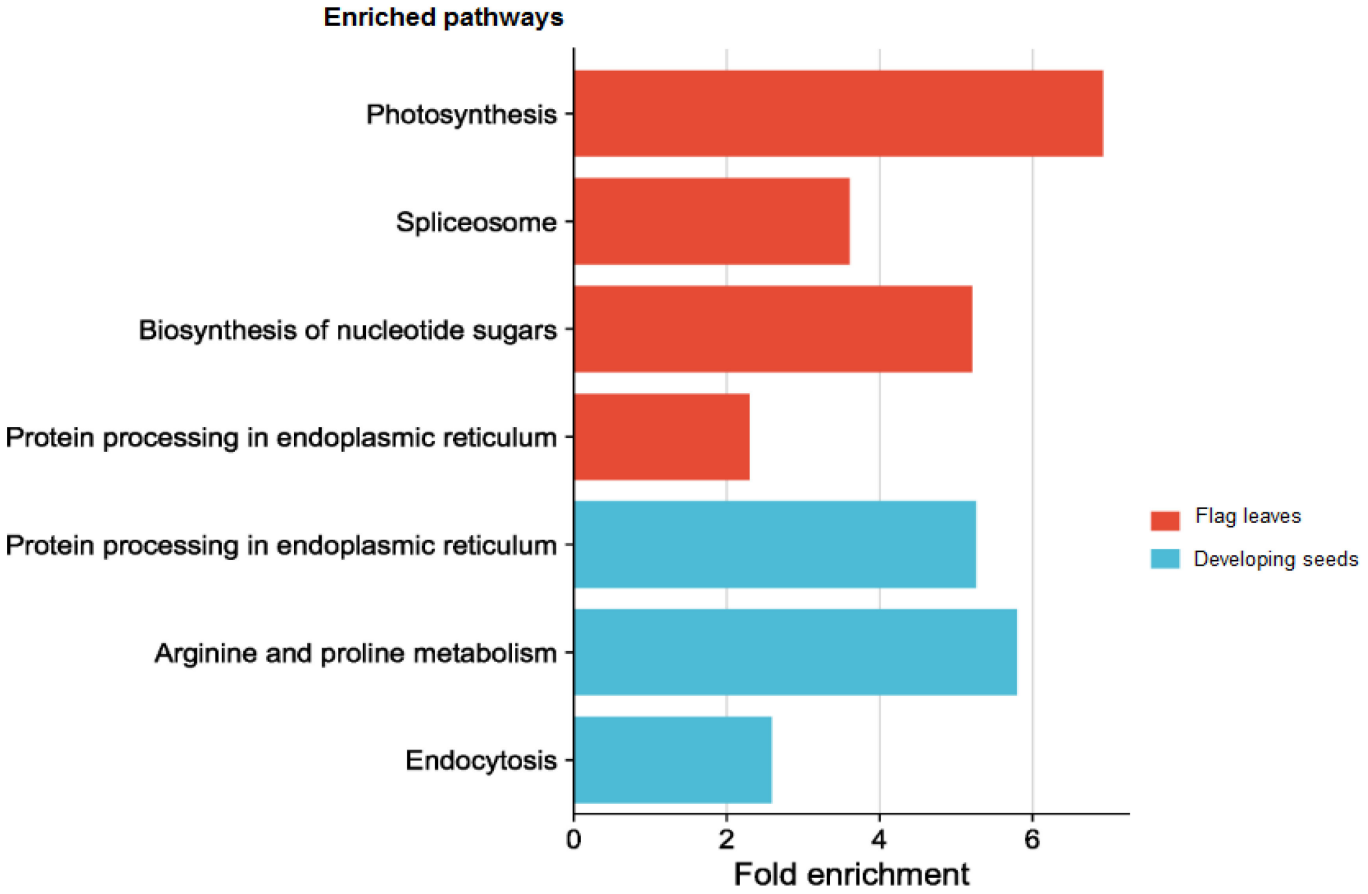
Top enriched pathways of the targets of the differentially expressed miRNAs in different tissues.

Due to the complicated role of miRNAs in the regulation of gene expression, a single or few DEMs cannot adequately depict the thorough functional distinction in the tissues under study. Hence GO and KEGG pathway enrichment analyses were done to present the functional distribution profiling of the target genes for the DEMs in the two tissues separately (**Supplementary Table S6**). As per the categorization of GO annotation of the DEMs in flag leaves, 18 biological processes (BP) were identified out of which the most frequent terms were translation (GO:0006412), protein folding (GO:0006457) and protein oligomerization (GO:0051259). The most frequent terms in cellular component (CC) included cytosol (GO:0005829), chloroplast thylakoid membrane (GO:0009535) and precatalytic spliceosome (GO:0071011).Metal ion binding (GO:0046872), unfolded protein binding (GO:0051082) and GTP binding (GO:0005525) were the top-represented terms in molecular function (MF).In developing seeds, 16terms of BP were identified, with the majority taking part in response to heat (GO:0009408),protein oligomerization (GO:0051259), and response to hydrogen peroxide (GO:0042542).15 CCGO classes were identified, with the most frequent GO terms being plasma membrane (GO:0005886), endoplasmic reticulum (GO:0005783) and plastid (GO:0009536).In MF, out of the 17 terms identified, the top three were associated with unfolded protein binding (GO:0051082), protein self-association (GO:0043621) and small GTPase binding (GO:0031267). In order to fully comprehend the specifics of target gene functions, gene ontology enrichment was performed. **Figures 8A, B** display the top 30 enrichment terms for each tissue. KEGG pathway enrichment analysis showed that the three pathways with the highest enrichment significance were photosynthesis, spliceosome and biosynthesis of nucleotide sugars in the flag leaves. In the developing seeds, the top enriched pathways were protein processing in the endoplasmic reticulum, arginine and proline metabolism and endocytosis (**Figure 9**; **Supplementary Table 7**), indicating potential tissue-specific heat stress response pathways. **Supplementary Table S6** shows the network plot for the miRNAs and their targets associated withtaes00195 (photosynthesis), taes03040 (spliceosome), taes01250 (biosynthesis of nucleotide sugars), and taes04141 (protein processing in endoplasmic reticulum) in flag leaf. Whereas for developing seeds, the network plot is shown in **Supplementary Figure S2B**.

### 3.5 Confirmation of differentially expressed miRNAs through qRT-PCR

**Figure 10:**
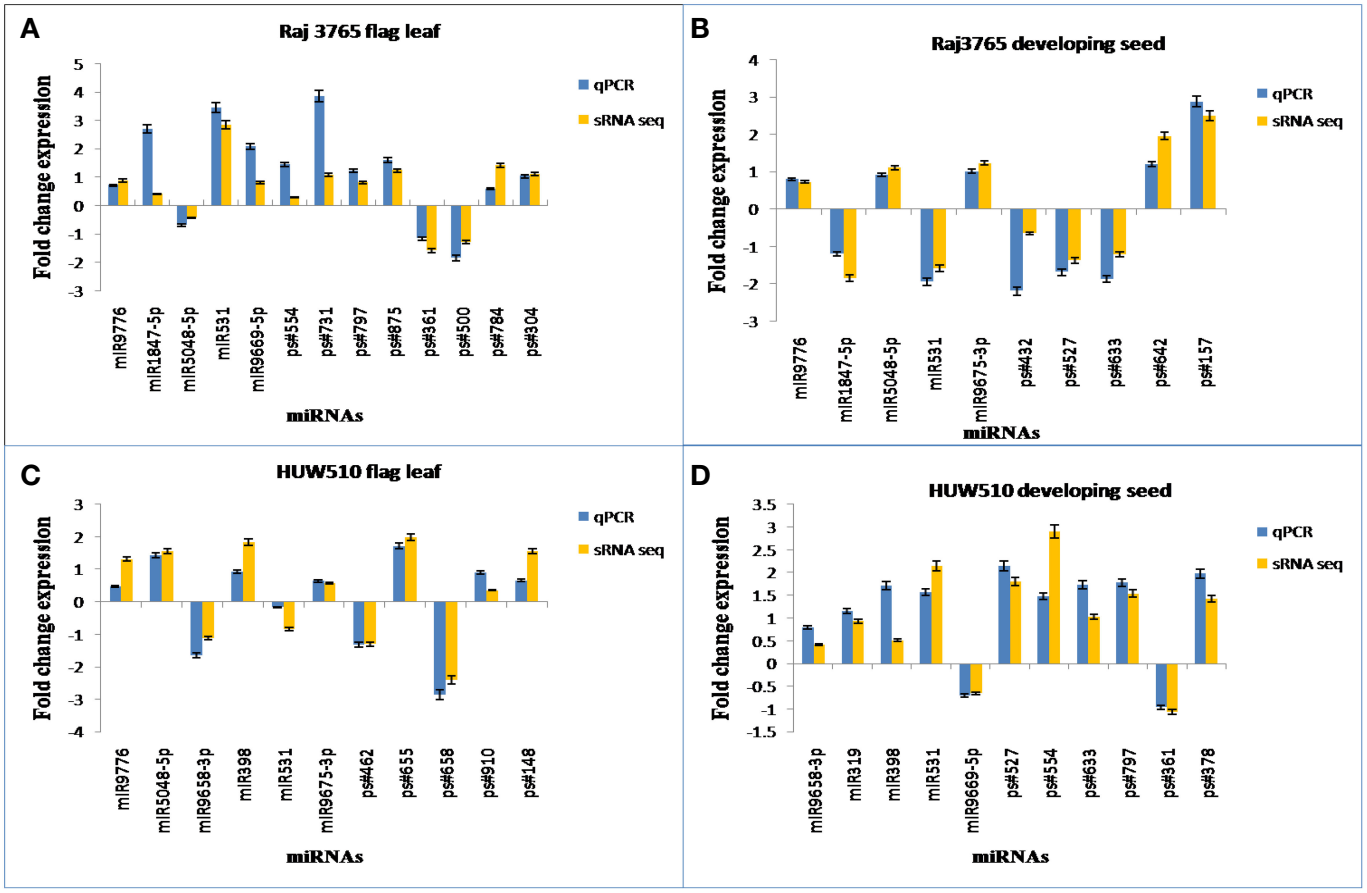
Experimental validation of known and novel miRNAs through qPCR. The sRNA-Seq log2FC (Fold Change) value and the qPCR log2FC of randomly chosen conserved and novel miRNAs in **(A)** RAJ3765 at flag leaf **(B)** RAJ3765 at developing seed **(C)** HUW501 at flag leaf **(D)** HUW510 at developing seed.

The expression patterns of the randomly selected known as well as novel miRNAs used for qPCR (**Supplementary Table S7**), corresponded well with the expression analysis data of small RNA sequencing (**Figure 10**).

## 4 Discussion

Abiotic stresses such as high temperatures detrimentally affect the growth, development and yields of wheat worldwide. In recent years, there have been considerable advances in the studies involving high-throughput sequencing analysis for the identification of plant miRNAs, their associated gene targets as well and the network pathways (Jones-Rhoades et al., 2006; Wu, 2013; Sun et al., 2019). In this study, we did an inclusive analysis of (1) identification of known and novel miRNAs in flag leaves and developing seeds of both genotypes (2) differential expression of tissue and genotype-specific miRNAs (3) Target identification via degradome and functional enrichment of the differentially expressed miRNAs in both the tissues.

Deep sequencing of the 8 sRNA libraries revealed 84 conserved miRNAs belonging to 35 miRNA families and 93 novel miRNAs (**Supplementary Tables S2**, **S3**). Most of these miRNAs were wheat-specific, but few were homologous to miRNAs in other monocots (**Supplementary Table S4**), suggesting a common evolutionary ancestry. In our study, the MIR1122 family had 7 members, the highest representation in terms of abundance of miRNAs (**Figure 2**). Similar findings were reported in wheat under various stress conditions (Kumar et al., 2014; Bakhshi et al., 2017; Sun et al., 2018; Zeeshan et al., 2021). Our study has confirmed many of the identified conserved miRNAs that have been reported earlier to be heat stress responsive e.g.tae-miR9664-3p, tae-miR9662a-3p, tae-miR1127a, tae-miR7757-5p, tae-miR9675-3p, tae-miR397-5p (Han et al., 2014), tae-miR167 (Chen et al., 2012; Kruszka et al., 2014; Ebrahimi Khaksefidi et al., 2015; Hivrale et al., 2016), tae-miR171 (Chen et al., 2012; Hivrale et al., 2016), tae-miR396 (Giacomelli et al., 2012; Hivrale et al., 2016).

### 4.1 miRNAs in flag leaves vs developing seeds

To better understand the distinct response among different organs to heat stress, we comprehensively compared the miRNA profiles in flag leaves and developing seeds. The two tissues showed variations in the known miRNA expression levels. e.g. tae-miR9672a-3pwas the most abundant among all the libraries, being more abundant in the flag leaves of the heat-stressed plants as compared to the developing seeds. Tae-miR9669-5p, tae-miR9774 and tae-miR5048-5p had higher number of reads in flag leaves as compared to the developing seeds in both the genotypes indicating their involvement in different physiological processes in these tissues as reported earlier by Han et al. (2014). For developing seed miRNAs, the HT genotype had a higher number of miRNAs commonly expressed in the treatment as well as the control groups than in the HS genotype. A similar trend was observed for the flag leaf too suggesting that the HT genotype had higher miRNA expression specificity for both tissues. Our results confirm the expression of tae-miR9664-3p in both flag leaf and developing seeds as suggested by Han et al. (2014).

In the flag leaves and developing seeds of the HT genotype, a wheat-specific miRNA, tae-miR9662a-3pwas found to be significantly induced in response to heat stress with a log-transformed fold change of 4.75 and 7.12, respectively. Degradome confirmed that miR9662a-3ptargeted a mitochondrial transcription termination factor (mTERF). Tae-miR9662a-3p has been reported in wheat under heat stress (Ravichandran et al., 2019) and seedling stages in wheat under drought stress (Li et al., 2019; Wang et al., 2021; Yue et al., 2022). Tae-miR5200 also showed a significant fold change of 5.47 in HT flag leaf, cleaving the transcript that codes for diacylglycerol kinase, a key enzyme of the lipid signaling pathway that mediates plant growth, development, and responses to biotic and abiotic cues (Rawat et al., 2021). Tae-miR1137b-5p cleaved serine-threonine kinase (STK), was found to be the most downregulated (7.0 ↓fold) after heat stress treatment in HT genotypes at FL stage as well as in HS-FL (8.0 ↓fold). STKs are generally involved in signal transduction pathways and cause phosphorylation of TFs namely bZIP, NAC and DREB (Kulik et al., 2011). In HS genotypes at FL stage, tae-miR9662b-3p was induced in response to high temperature, regulating transcripts coding for NB-ARC domain-containing protein. It has been reported to be heat-responsive in wheat by Goswami et al. (2014). Tae-miR1136 was down-regulated in all the samples after heat stress.

Tae-miR9666b-3pwas the most downregulated (6.1 ↓fold) in the developing seeds of the HT genotype. It was found to target endoglucanase involved in starch and sucrose metabolism. It has been found that tae-miR9666b-3p is potentially involved in developing grains in response to nitrogen levels (Hou et al., 2020) and also in response to salinity (Zeeshan et al., 2021), but there are no reports of its involvement in heat stress so far.

Some of the miRNAs showed contradictory expression within the tissues which could be due to their possible roles in different physiological processes in these tissues e.g. Tae-miR9677a, targeting the AGO (argonaute) was upregulated in flag leaves whereas downregulated in the seeds of the HT genotype after the heat stress treatment. Similarly, tae-miR5384-3p was downregulated in flag leaves and upregulated in seeds of the HT genotype in response to heat stress. Tae-miR5384-3p targeted the heat shock cognate 70 kDa protein gene. It is a cofactor of HSP70 that serves as a molecular chaperone. These play a key role in the stabilization, folding and translocation of newly synthesized proteins. The interactions between plant heat tolerance and HSP70 have been reported in *Arabidopsis* (Kim and Schöffl, 2002), wheat (Pandey et al., 2015; Kaur et al., 2016), rice (Wahab et al., 2020), barley (Chaudhary et al., 2019), maize (Jiang et al., 2021) etc. The Tae-miR5384-3p has been shown to be involved in response to abiotic stress in wheat (Singh and Mukhopadhyay, 2021) and sugarcane (Selvi et al., 2021).

As per GO (Gene Ontology) enrichment analysis of the target genes of DEMs in FL, the top two GO terms were UDP sulfoquinovose synthase activity and LSM1-7-Pat1 complex. The soluble enzyme UDP-sulfoquinovose synthase is found in the chloroplast stroma where it regulates the synthesis of the sulfolipid that stabilizes protein complexes like photosystem II (Minoda et al., 2003). The genes involved in secondary metabolism such asUDP-sulfoquinovose synthase have been reported to decrease under heat stress (Shi et al., 2017). In *Arabidopsis thaliana*, the Lsm1–7 complex has been shown to act as an activator of decapping, where it interacts with a variety of stress-inducible transcripts, selecting them for decapping and eventual destruction during abiotic stress (Perea-Resa et al., 2016). This interaction guarantees the target transcripts’ proper turnover, which in turn provides the downstream stress-responsive gene expression, particularly ABA biosynthesis which is necessary for plant adaptation to abiotic stress (Suzuki et al., 2016). Based on the KEGG pathway enrichment analysis, the targets of these DEMs showed a major involvement inthephotosynthetic pathway (taes00195), biosynthesis of nucleotide sugars (taes01250), spliceosome pathway (taes03040) and protein processing in the endoplasmic reticulum (taes04141) in the flag leaf. The first two pathways are related to biosynthetic functions and the rest two are related to membrane functions; functions that are strongly tied to the resilience of plants to environmental heat stress. Photosynthesis is the prime target of all abiotic stresses as the leaves help manage the survival of the plant under stress. The miRNAs tae-miR1137a, tae-miR9653a-3p, tae-miR9653b, ps#793 and tae-miR530 were involved in the photosynthesis pathway, targeting the transcripts photosystem II protein, photosystem II reaction center W- protein, ATP synthase subunit b and oxygen-evolving enhancer protein 2. The ultimate energy source for the synthesis of carbohydrates, lipids, peptides, and secondary metabolites is provided by the nucleotide sugars, which are created from the carbohydrates produced during photosynthesis (Stasolla et al., 2003). As photosynthesis is affected by increased temperatures, the pathways involved in sugar metabolism are bound to change as a result of it, thereby affecting the overall growth and development of crops.

Splicing and/or alternative splicing (AS) play highly significant roles in controlling the miRNA levels in stress conditions in plants (Ling et al., 2017). AS increases proteome diversity and controls post-transcriptional gene expression (Simpson et al., 2008). Yang et al. (2012) demonstrated that spliceosome activity is linked to the regulation of certain miRNA-target interactions in *A. thaliana.* Similar findings were reported in barley where heat stress led to the AS of (pri-miRNAs) of miR160a and miR5175a, resulting in the accumulation of mature miRNAs (Kruszka et al., 2014). Further exploration is necessary to better understand the complexity of wheat miRNA expression, spliceosomal protein functions, and the synthesis of pri-miRNA isoforms.

In developing seeds, the GO enrichment analysis showed HSP70 protein binding, protein self-association and protein oligomerization, response to hydrogen peroxide and heat as the topmost enriched terms. The results show an interaction between the synthesis of HSP and ROS, demonstrating that ROS can induce HSPs as signaling molecules, supporting earlier results (Timperio et al., 2008; Shah et al., 2011). KEGG pathway enrichment analysis of the targets of the differentially expressed miRNAs showed that in the developing seeds, the top enriched pathways comprised of protein processing in endoplasmic reticulum (taes04141), arginine and proline metabolism(taes00330) and the endocytosis pathway (taes04144).The endoplasmic reticulum (ER) facilitates efficient protein synthesis, maturation, and secretion. Unfolded and misfolded proteins can build up in the ER lumen as a result of severe environmental conditions, which can result in ER stress and affect the ER quality control machinery (Eichmann and Schafer, 2012). ER also plays an important role in the metabolism under heat stress, specifically in the starch and lipid biosynthesis pathways. Plants modify the lipid composition of membranes to counteract heat-induced membrane instability, which calls for strict management of lipid metabolism under heat stress. Many studies have implicated the involvement of ER in “chalky” grain appearance and decreased starch content in rice. Also, altered expression of starch biosynthetic genes in mutants of the UPR, particularly in seeds has been shown (Yasuda et al., 2009). Recently three ER stress-responsive miRNAs, tae-miR164, tae-miR2916, and tae-miR396e-5p were identified in a study, where knockdown of these miRNAs resulted in increased tolerance of wheat plants to heat, drought and salt stress (Chen and Yu, 2023). Endomembrane trafficking is strongly linked to stress signaling pathways in order to fulfill the demands of rapid changes in cellular processes and ensure the correct delivery of stress-related cargo molecules (Wang et al., 2020). In our study, tae-miR1127a targeted the AP2 adaptor complex, a multimeric protein found on the cell membrane that helps import cargo during clathrin-mediated endocytosis. Many heat responsive proteins take part in clathrin-mediated endocytosis, and these have been shown to regulate the abundance as well as distribution of signaling receptors and transporters due to heat stress (Paez Valencia et al., 2016). Plants maintain cellular homeostasis by accumulating osmoregulatory metabolites such as proline and arginine. Heat stress produces a large amount of ROS, and the synthesis of peroxisomes, arginine, and proline can effectively remove excess ROS in cells and avoid oxidative damage to the plant (De Leonardis et al., 2015). In our study, tae-miR5175-5p, tae-miR1122b-3p, tae-miR1137a, tae-miR1127a, and tae-miR1137b-5p were involved in arginine and proline metabolism in the developing seeds samples.

### 4.2 miRNAs in heat tolerant vs susceptible genotypes

Comparison of the temporal expression profiles of miRNAs in the heat tolerant vs. heat susceptible genotypes after heat stress reveals variation in the relative accumulation of miRNAs between the two genotypes. Under normal conditions, 92 miRNAs were differentially expressed in flag leaves of the HT and HS genotypes, while the number increased to 99 after heat stress exposure. In the developing seeds, the number of differentially expressed miRNAs reduced to 96 after heat stress exposures, which were 100 in non-stressed samples. This clearly indicates that the contrasting responses of the HT and HS genotypes during heat stress conditions might be associated with the molecular events that arise due to differences in the miRNA expression and regulation and thus result in the variation of their tolerance to heat stress.

In the FL of HT vs HS genotypes, 48 miRNAs were commonly expressed in both the samples, among which tae-miR9674b-5p, tae-miR9663-5p, tae-miR9662b-3p were the most upregulated (7↑, 4.8↑, 4.4↑folds) and tae-miR9674a-5p, tae-miR156, tae-miR9679-5p were the most downregulated (8.9↓, 8.2↓, 7.4↓folds) in response to heat in FL of the two genotypes. In the developing seeds of both the genotypes, 51 miRNAs were common to both the libraries. Tae-miR167c-5p, tae-miR5084, tae-miR9666b-3p were the most upregulated (7.6↑, 5.7↑, 5.3↑ folds) while tae-miR1118, tae-miR1122a, tae-miR7757-5p were most downregulated after heat stress.

In our study, tae-miR167c-5p showed genotype-specific expression pattern after heat stress. It was downregulated in both the tissues of the HT genotype and these targeted the phytohormone ARF. Similar expression patterns of the MIR167 family have been shown in barley (Kruszka et al., 2014), rice (Saibo et al., 2009; Sailaja et al., 2014) and wheat as well (Kumar et al., 2015; Ragupathy et al., 2016). In rice, osa-miR167 regulates the auxin-miR167-ARF8-OsGH3.2 pathway, which functions during grain filling (Xue et al., 2009). In contrast to this, tae-miR167c-5p was upregulated in the developing seeds of the HS genotype. The differentially expressed miRNAs between superior and inferior wheat grains are likely related to cell division, carbohydrate metabolism and hormone biosynthesis (Wang et al., 2018). Tae-miR160 also showed down regulation in flag leaf as well as developing seeds. Tae-miR160 was reported to be downregulated in response to heat stress (Goswami et al., 2014), salt stress (Zeeshan et al., 2021) and nitrogen stress as well (Sinha et al., 2015), indicating its relevance in abiotic stresses.

Tae-miR9666a-3p was downregulated in response to heat stress in the developing seeds of the HS genotype while it was upregulated in the HT genotype. It targeted the DNA-directed RNA polymerase subunit (EC 2.7.7.6). Tae-miR9666a-3p has been reported to be involved in regulating grain characteristic levels in wheat (Hou et al., 2020). In our study, its converse expression in the contrasting genotypes indicates its role in seed development under high-temperature conditions. These findings suggest that genotypes with different heat sensitivities have distinct mechanisms for miRNA regulation of heat tolerance.

Many novel miRNAs also had a relatively high abundance, and their expression levels changed significantly after heat stress e.g. ps#15 showed a 1.8-fold change in the HT and 1.7-fold in HS developing seeds. Degradome results showed that it targeted MAP kinase, which is activated by heat stress-induced Ca^2+^ influxes and ROS (Sangwan et al., 2002). The number of known and novel miRNAs in flag leaves and developing seeds as well as in the two wheat genotypes under control and stressful conditions demonstrated their functions in imparting stress tolerance to heat.

## 5 Conclusions

This study provides a comprehensive comparative analysis and specific miRNA profile of heat-induced miRNAs in two tissues of contrasting genotypes under heat stress. The comparative analysis clearly showed that different tissues of heat-tolerant and susceptible wheat genotypes have different expression profiles under different conditions. This might be associated with the molecular events leading to contrasting responses of these genotypes to high temperatures. Possible target genes were identified for the differentially expressed miRNAs using degradome analysis, GO and KEGG annotations. The miRNAs were discovered to be associated with abiotic stress tolerance- protein processing (HSPs), photosynthesis (photosystem proteins), spliceosome etc. The identification of tissue and genotype preferential miRNAs will help in widening the knowledge of the molecular basis of thermotolerance in wheat and other plants as well.

## Data availability statement

The data presented in the study are deposited in the National Center for Biotechnology Information Databank (NCBI) (accession number PRJNA1012670 and PRJNA1013021).

## Author contributions

MS: Data curation, Formal analysis, Methodology, Writing – original draft. AA: Visualization, Writing – review & editing. GS: Funding acquisition, Writing – review & editing. PS: Conceptualization, Investigation, Project administration, Resources, Supervision, Writing – review & editing.
