# Supplementary material for: Genome-wide expression analysis of novel heat-responsive microRNAs and their targets in contrasting wheat genotypes at reproductive stage under terminal heat stress
Source: Front Plant Sci. 2024 Apr 10;15:1328114. doi: 10.3389/fpls.2024.1328114 (PMC11039868; doi:10.3389/fpls.2024.1328114)

Supplementry Figure S1. Secondary structures representation of a few novel miRNAs precursors.

(A) ps#774

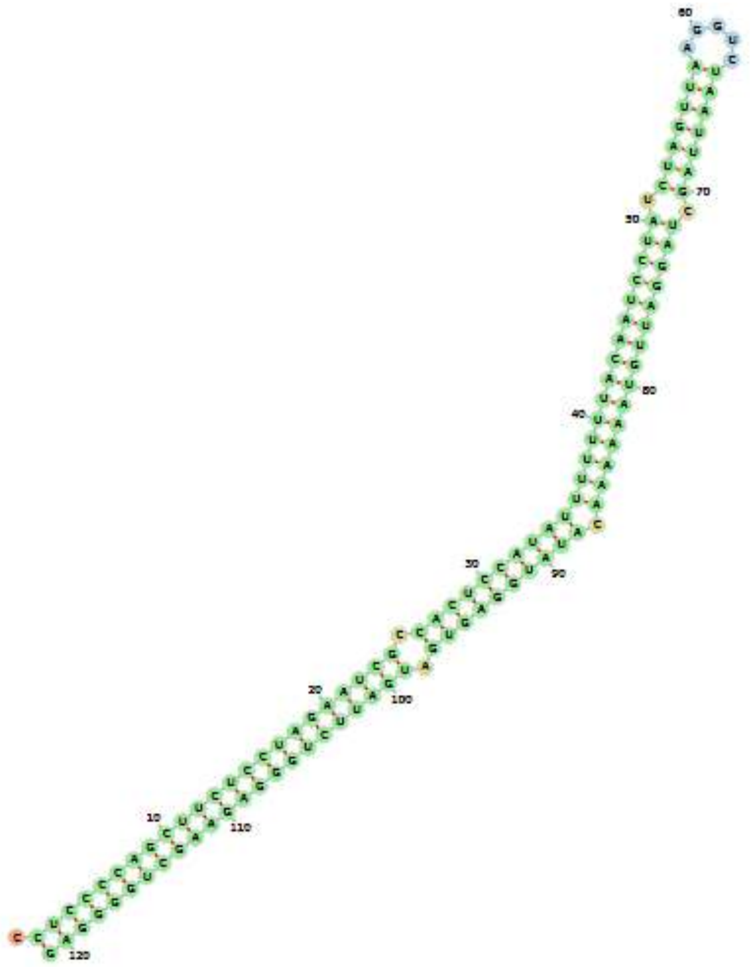

(B) ps#658

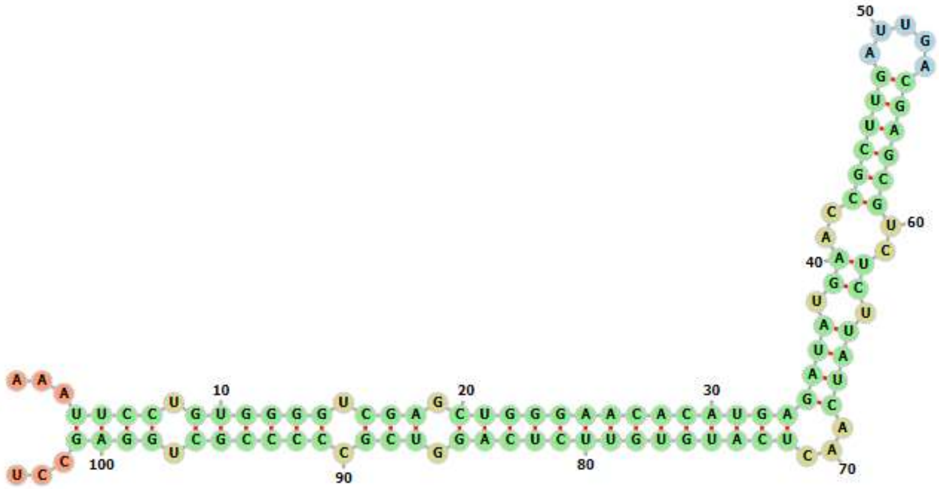

(C) ps#793

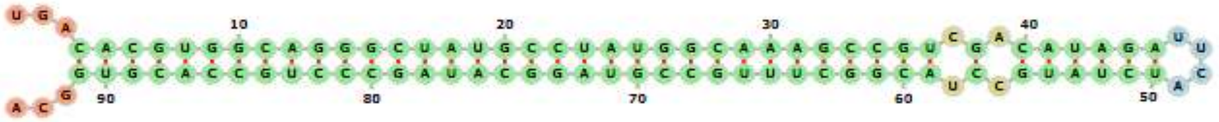

(D) ps#242

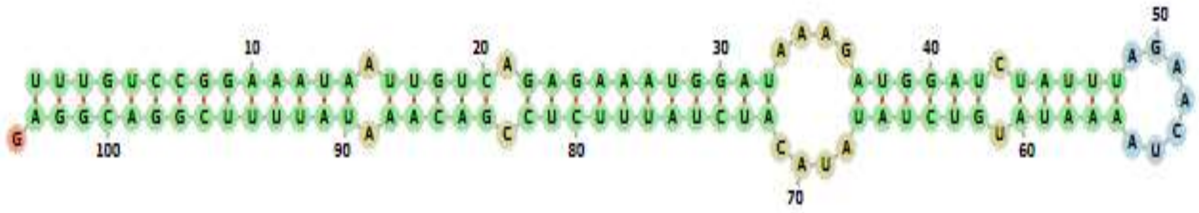

(E) ps#378

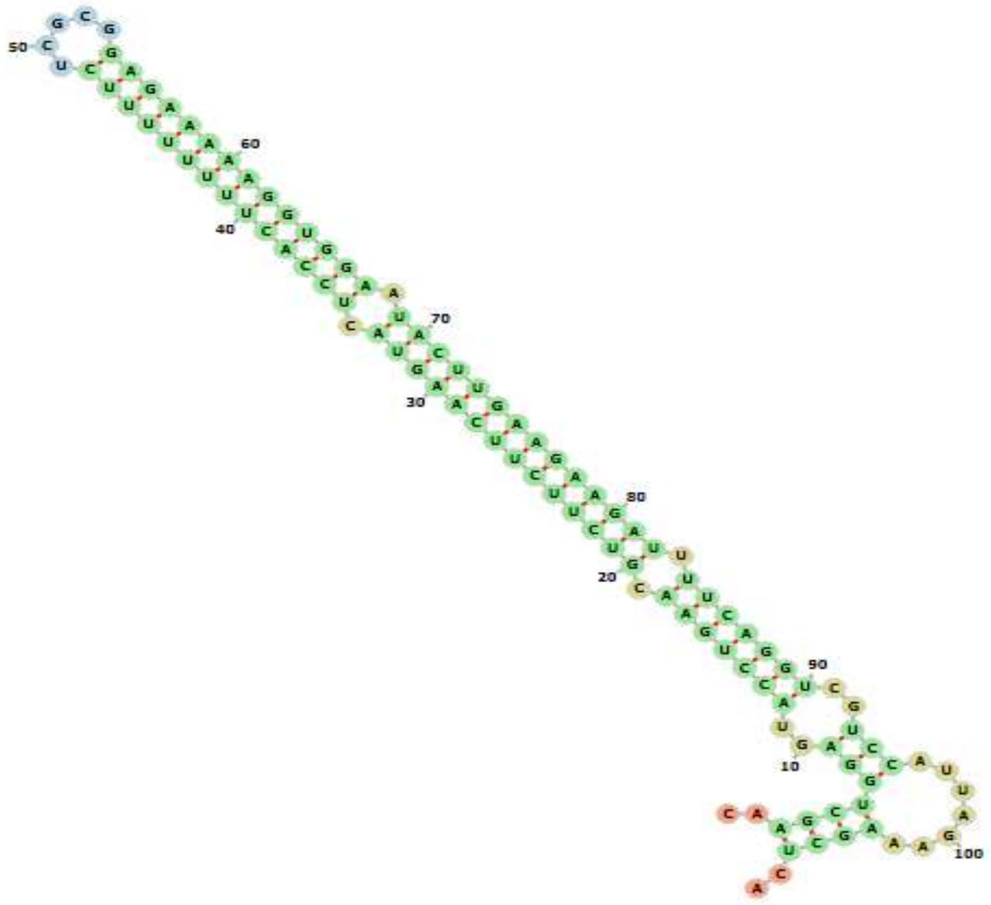

(F) ps#12

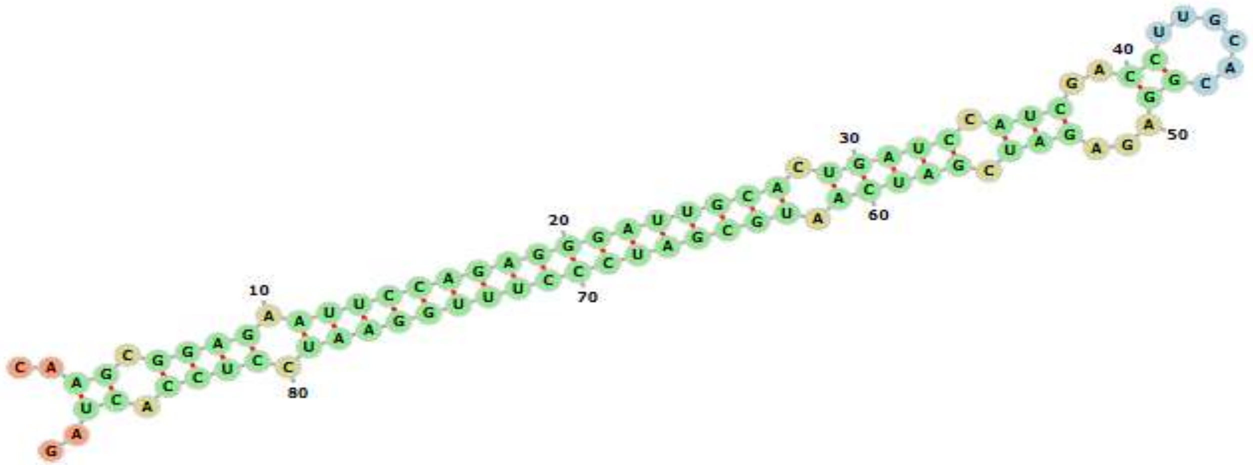

Supplement: Supplementary file 1 [file Image_1.pdf]
