# Supplementary material for: Genome-wide expression analysis of novel heat-responsive microRNAs and their targets in contrasting wheat genotypes at reproductive stage under terminal heat stress
Source: Front Plant Sci. 2024 Apr 10;15:1328114. doi: 10.3389/fpls.2024.1328114 (PMC11039868; doi:10.3389/fpls.2024.1328114)
Supplement: Supplementary file 2 [file Image_2.pdf]

Supplementry Figure S2**.** Network plot for the miRNAs and their targets associated withtaes00195 (Photosynthesis), taes03040 (Spliceosome), taes01250 (Biosynthesis of nucleotide sugars), and taes04141 (Protein processing in endoplasmic reticulum) in flag leaf **(A)**; taes04141 (Protein processing in endoplasmic reticulum), taes00330 (Arginine and proline metabolism), and taes04144 (Endocytosis) in developing seeds **(B)**.

(A)

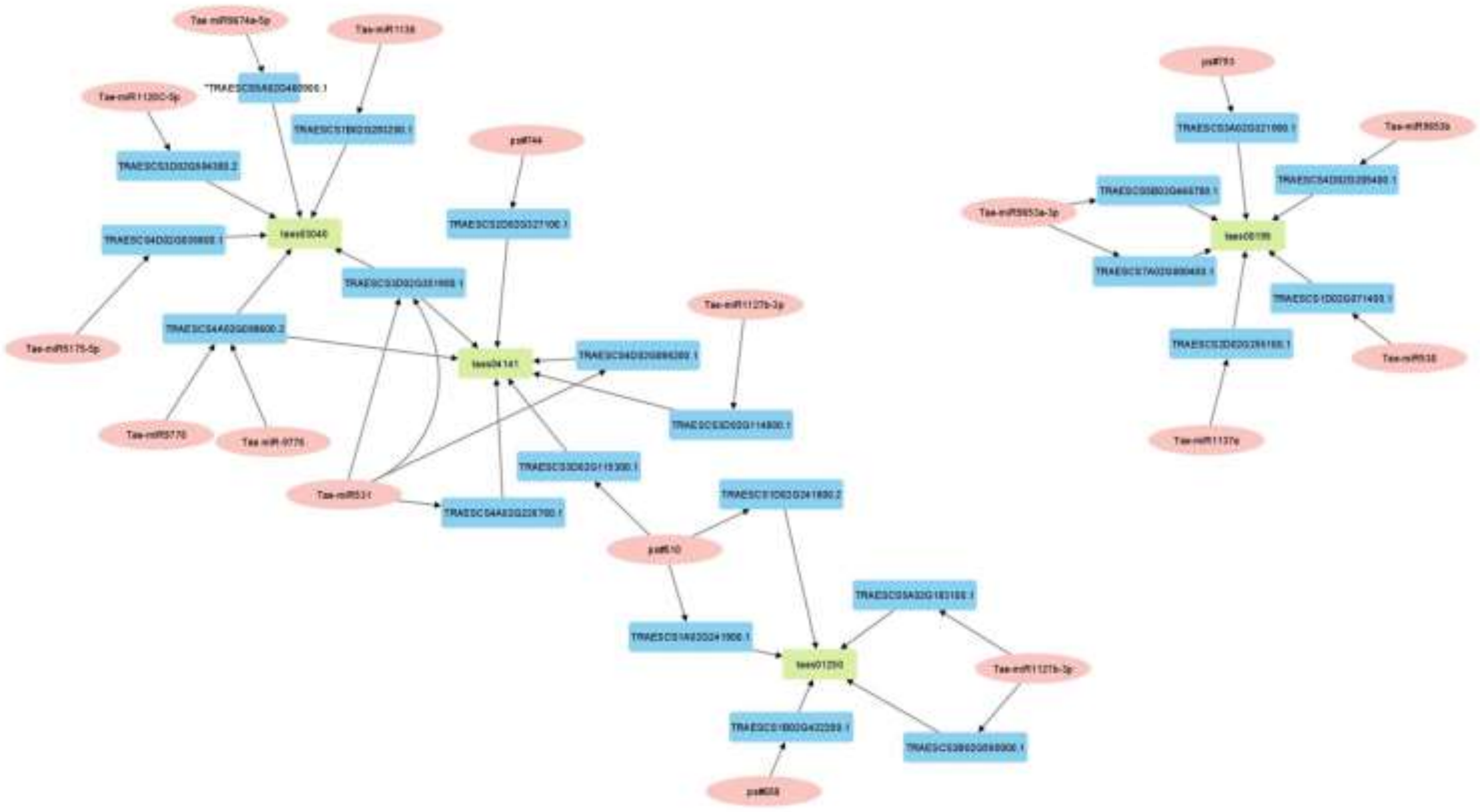

(B)

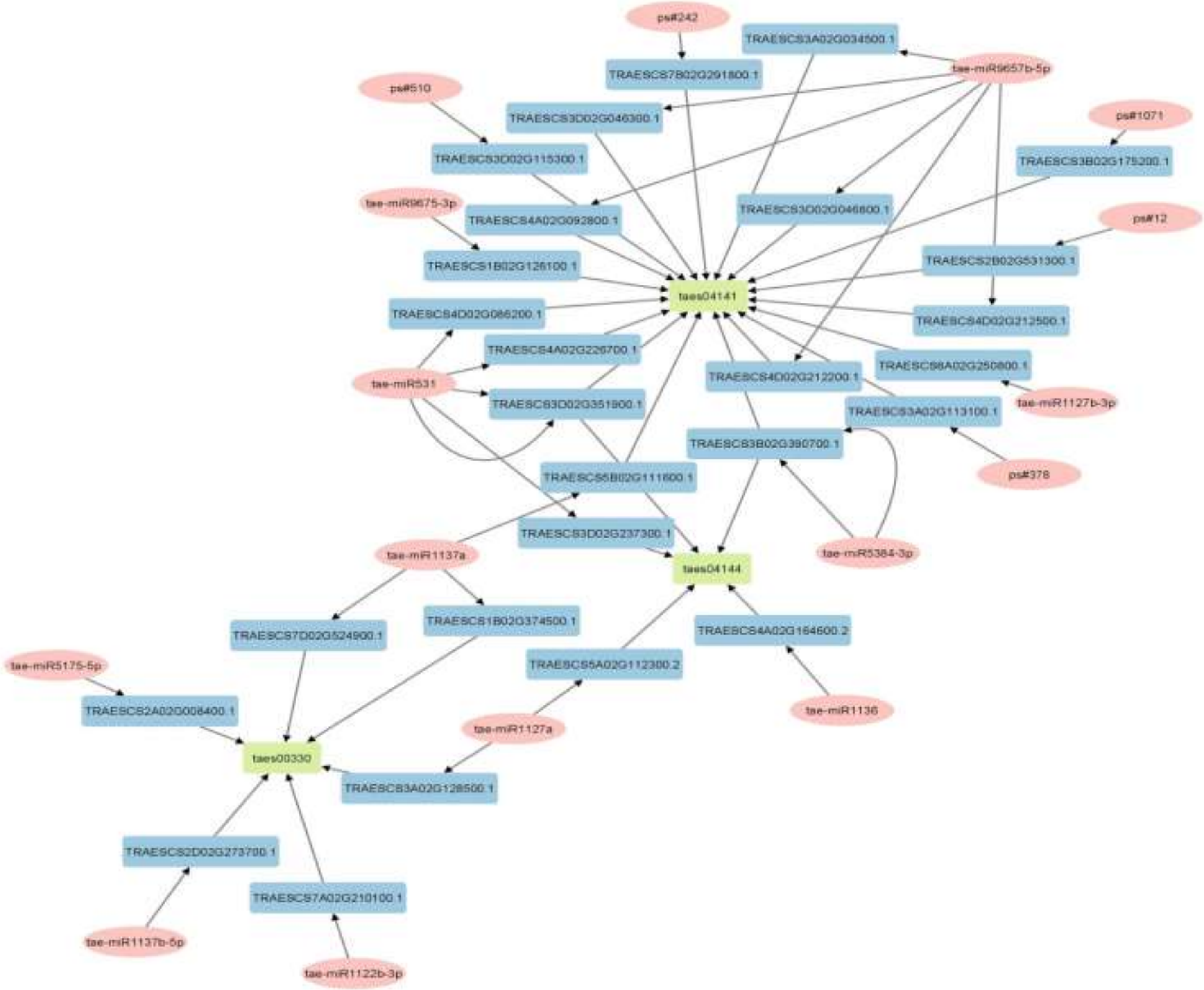

ps#242
TRAESCS3A02G034500.1
TRAESCS7B02G291800.1
tae-miR9657b-5p
ps#510
TRAESCS3D02G046300.1
ps#1071
TRAESCS3D02G115300.1
TRAESCS3B02G175200.1
tae-miR9675-3p
TRAESCS4A02G092800.1
TRAESCS3D02G046800.1
ps#12
TRAESCS1B02G126100.1
TRAESCS2B02G531300.1
taes04141
TRAESCS4D02G086200.1
TRAESCS4D02G212500.1
TRAESCS4A02G226700.1
TRAESCS6A02G250800.1
tae-miR531
TRAESCS4D02G212200.1
tae-miR1127b-3p
TRAESCS3D02G351900.1
TRAESCS3A02G113100.1
TRAESCS3B02G390700.1
ps#378
TRAESCS5B02G111600.1
TRAESCS3D02G237300.1
tae-miR1137a
tae-miR5384-3p
taes04144
TRAESCS1B02G374500.1
TRAESCS7D02G524900.1
TRAESCS4A02G164600.2
tae-miR5175-5p
TRAESCS5A02G112300.2
TRAESCS2A02G008400.1
tae-miR1136
tae-miR1127a
taes00330
TRAESCS3A02G128500.1
TRAESCS2D02G273700.1
TRAESCS7A02G210100.1
tae-miR1137b-5p
tae-miR1122b-3p
